# Role of functional fatty acids in modulation of reproductive potential in livestock

**DOI:** 10.1186/s40104-022-00818-9

**Published:** 2023-02-14

**Authors:** Xiangzhou Zeng, Siyu Li, Lu Liu, Shuang Cai, Qianhong Ye, Bangxin Xue, Xinyu Wang, Shihai Zhang, Fang Chen, Chuanjiang Cai, Fenglai Wang, Xiangfang Zeng

**Affiliations:** 1 State Key Laboratory of Animal Nutrition, Ministry of Agriculture Feed Industry Center, China Agricultural University, 100193 Beijing, P. R. China; 2 Beijing Key Laboratory of Bio feed Additives, 100193 Beijing, P. R. China; 3 State Key Laboratory of Agricultural Microbiology, College of Animal Sciences and Technology, Huazhong Agricultural University, 430070 Wuhan, Hubei China; 4 Guangdong Provincial Key Laboratory of Animal Nutrition Control, College of Animal Science, South China Agricultural University, 510642 Guangzhou, China; 5 College of Animal Science and Technology, Northwest A&F University, 712100 Yangling, Shaanxi China

**Keywords:** Embryo development, Fatty acids, Lactation, Oocyte, Placental, Pregnancy, Reproduction

## Abstract

Fatty acids are not only widely known as energy sources, but also play important roles in many metabolic pathways. The significance of fatty acids in modulating the reproductive potential of livestock has received greater recognition in recent years. Functional fatty acids and their metabolites improve follicular development, oocyte maturation and embryo development, as well as endometrial receptivity and placental vascular development, through enhancing energy supply and precursors for the synthesis of their productive hormones, such as steroid hormones and prostaglandins. However, many studies are focused on the impacts of individual functional fatty acids in the reproductive cycle, lacking studies involved in deeper mechanisms and optimal fatty acid requirements for specific physiological stages. Therefore, an overall consideration of the combination and synergy of functional fatty acids and the establishment of optimal fatty acid requirement for specific stages is needed to improve reproductive potential in livestock.

## Introduction

The health and reproductive performance of livestock are directly affected by maternal nutritional and physiological condition throughout gestation and lactation [1]. Nutrient shortages and excesses can cause reproductive disturbance and influence reproductive performance [2, 3]. The reproductive performance of livestock can be effectively improved by precisely satisfying the nutrient requirements of livestock at each reproductive stage [4]. Precise feeding patterns of functional amino acids during pregnancy have been found to benefit livestock placental and embryo development [5–9]. Recent research on fatty acids has shown that lipid metabolism is vital throughout the reproductive cycle [10]. Lacking or excessive maternal lipid reserves will cause a slew of complications, including delayed estrus, poor oocyte quality, low fertilization rates, abortion, intrauterine growth restriction (IUGR) and preterm birth in both mouse and human [11, 12]. In vitro and in vivo investigations, furthermore, have indicated that some functional fatty acids, primarily essential fatty acids, are involved in follicular development, oocyte maturation, endometrial receptivity, placental development, embryo development and lactation performance [13–15]. Therefore, adding appropriate fatty acids according to the change of reproductive cycle may effectively boost the livestock reproductive potential.

Fatty acids are now widely considered to play 4 major physiological roles. First, fatty acids can be used as fuel molecules, and those mobilized in the form of triacylglycerols are oxidized to provide energy for cells and organisms. Second, fatty acids are components of phospholipids and glycolipids. These amphipathic molecules play a crucial role in biological membranes. Third, many proteins are modified by the covalent attachment of fatty acids, allowing them to be targeted to membranes. Fourth, fatty acid derivatives also function as hormones and intracellular messengers [16]. So, a substantial amount of research has been conducted on fatty acid nutrition in livestock, and as a result, the significance of essential fatty acids in livestock reproduction has received increasing attention. Because of their distinct activities in metabolic regulation and physiology, nonessential long-chain fatty acids as well as short- and medium-chain fatty acids have begun to receive greater attention in recent years. Studies on fatty acid nutrition have gradually shifted focus away from whether or not they are essential fatty acids to their functions. Recently, a new concept of functional fatty acids has arisen, which is defined as fatty acids participating in and regulating key metabolic pathways of biological health, development, growth and reproduction. In this review, the molecular mechanisms by which functional fatty acids improve follicular development, promote oocyte maturation and embryo development by improving energy status. and increasing precursors of synthetic reproductive hormones, such as steroids and prostaglandins (PGs). And increasing precursors of synthetic reproductive hormones, such as steroids and prostaglandins (PGs), are reviewed. Finally, the roles of fatty acids and their metabolites in endometrial receptivity, placental vascular development and lactation are analyzed. Moreover, the molecular mechanisms by which functional fatty acids and their metabolites affect livestock reproductive performance are explored in depth, thereby explore the potential for functional fatty acids to improve the reproductive performance in modern farming and animal husbandry.

## Metabolism of functional fatty acids in livestock

### Sources and synthesis of functional fatty acids in livestock

**Fig. 1:**
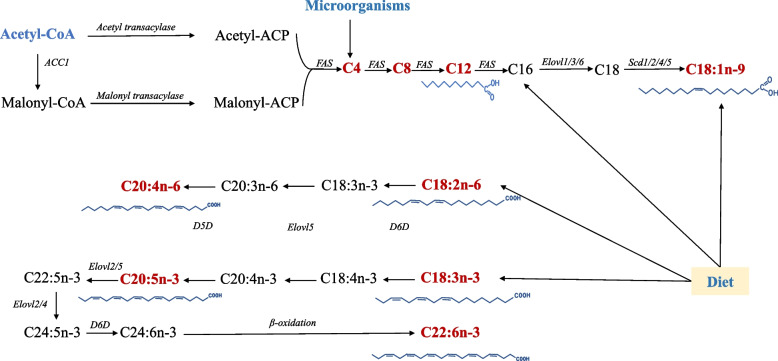
Pathways of endogenous and dietary fatty acid synthesis of functional fatty acids in livestock. ACC1: Acetyl-CoA carboxylase 1; FAS: Fatty acid synthase; Elovls: Elongase of very long chain fatty acids; Scd: Stearoyl-CoA desaturase; D5D: Δ5 desaturase; D6D: Δ6 desaturase

Functional fatty acids are derived from endogenous synthesis in vivo or from diets on the length of the carbon chain and the number and position of unsaturated bonds. In livestock, nonessential fatty acids of functional fatty acids can be generated through the de novo synthesis of fatty acids, and because of the lack of enzymes for the synthesis of essential fatty acids of functional fatty acids, the synthesis of some fatty acids depends on the essential fatty acids obtained from food (Fig. 1). As indicated in the figure, the synthesis of fatty acids begins with the carboxylation of acetyl-CoA into malonyl-CoA, and the extension phase of fatty acid synthesis begins with the formation of acetyl-acyl carrier protein (acetyl-ACP) and malonyl-ACP by acetyl-CoA and malonyl-CoA under the catalysis of corresponding enzymes. Then through condensation, the two form acetoacetyl-ACP, which is then reduced, dehydrated and reduced to butyryl-ACP. Another cycle begins with the condensation of butyl-ACP and malonyl-ACP [16]. This series of reactions is repeated until the final product palmitate (C16) is formed, during which functional short- and medium-chain fatty acids are generated. Then, the carbon chain is extended based on the elongation of very long chain fatty acids (ELOVLs), and unsaturated bonds are formed by the stearoyl-CoA desaturase (SCD) family. The above synthesis process is mostly found in the liver of livestock, and microbes in the hindgut can also produce short-chain fatty acids.

Another way for livestock to obtain functional fatty acids is diet. Foraging is the primary source of nutrients for livestock to maintain growth and production, and the composition and number of fatty acids in diet have an impact on livestock growth, immunity and reproduction. Table 1 lists the distribution and key sources of some functional fatty acids in feed. Different livestock species have vastly diverse digestive tracts; therefore, absorption of fatty acids from diet varies across species as well. The fatty acid concentrations in the diet for ruminants are normally low [2.5%–3.5% of dry matter intake (DMI)] in the diet [17]. Oilseeds, various plants, and animal by-products containing fat or oil, such as fishmeal and distilled grain, are the most prevalent sources of lipids to cattle. The extent of the biohydrogenation can, however, be reduced in supplementary feeds by the use of protected oils such as calcium soaps which bypass the rumen and release long-chain polyunsaturated fatty acids (LCPUFAs) into the small intestine [18]. In monogastric animals, such as swine, a slightly higher proportion of fats and oils can be added directly to their feeds, which is often simply a way to increase the energy concentration in traditional diet formulas. The further synthesis of fatty acids in different livestock is nearly identical despite the differences in their sources, as shown in Fig. 1; livestock can synthesize other long-chain functional fatty acids: arachidonic acid (ARA), docosahexaenoic acid (DHA) and eicosadienoic acid (EPA) by alpha-linolenic acid (ALA) and linoleic acid (LA) in diet. Research over the last two decades has revealed that it is beneficial to use special fat sources that have not only high energy but also positive effects on reproduction and other physiological processes [7, 19, 20].

**Table 1:** Distribution and sources of functional fatty acids in diets [21–23]

Name	Number of carbon chain	Fatty acid type	Essential fatty acid	Percentage of oils commonly used in several diets, %				Main source
				**Soybean oil**	**Corn oil**	**Peanut oil**	**Sesame oil**	
Butyrate	4	Short chain	No	-	-	0.1	-	Milk, synthetic
Caprylic acid	8	Medium chain	No	-	-	-	-	Coconut oil, synthetic
Lauric acid	12	Medium chain	No	-	-	-	-	Coconut oil, synthetic
Oleic acid	18	Monounsaturated	No	23.7	1.9	42.9	4.8	Olive oil
Linoleic acid	18	Polyunsaturated	Yes	52.8	31.4	34.8	39.3	Safflower oil
α-Linolenic acid	18	Polyunsaturated	Yes	5.8	51.9	0.1	41.3	Corn oil
Arachidonic acid	20	Polyunsaturated	Yes	0.2	0.7	1.1	0.3	Meat, dairy
Eicosapentaenoic acid	20	Polyunsaturated	No	-	0.3	-	-	Fish oil
Docosahexaenoic acid	22	Polyunsaturated	No	-	-	-	-	Fish oil

### Digestion, absorption and metabolism of functional fatty acids in livestock

**Fig. 2:**
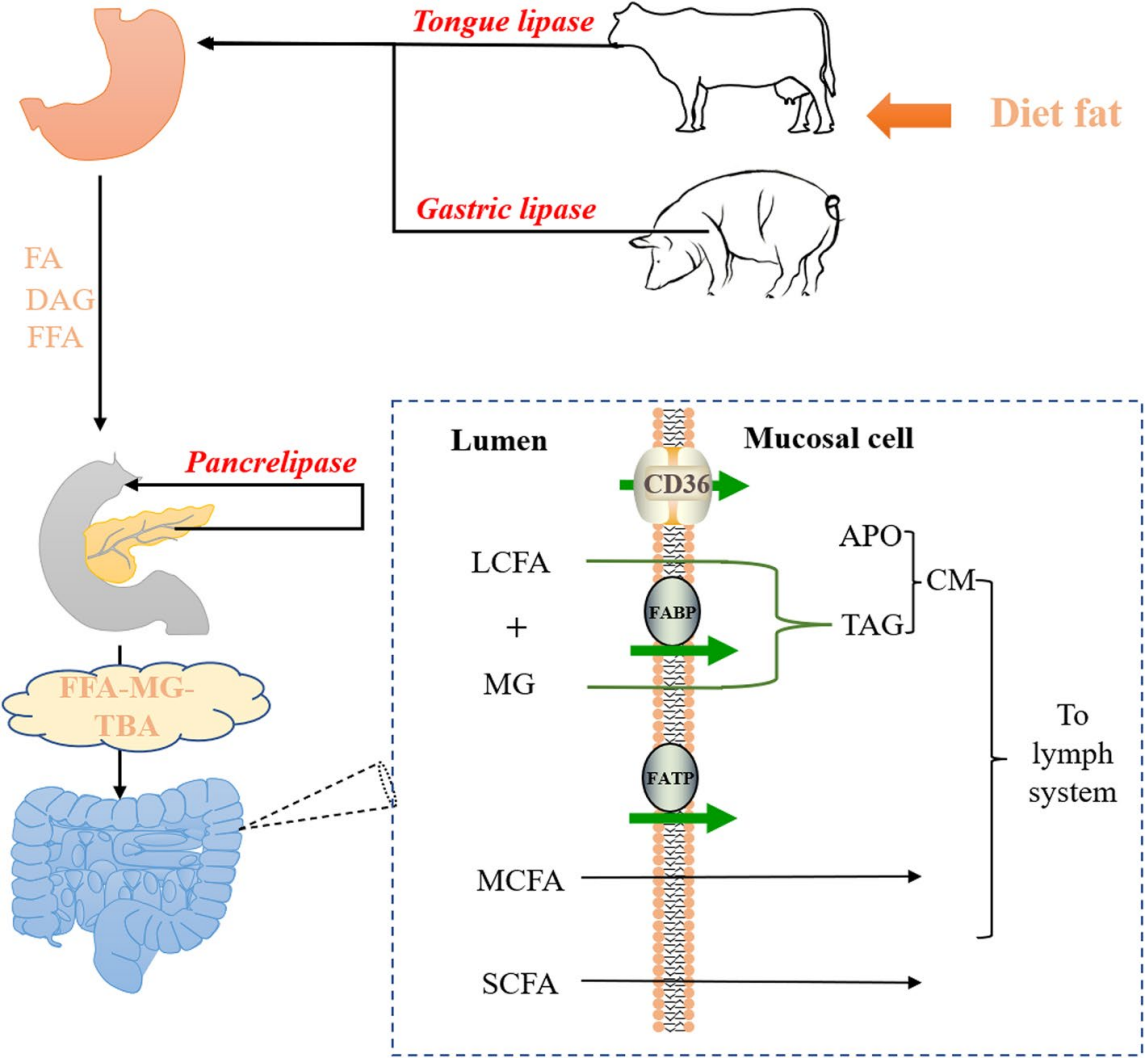
Fatty acid digestion and absorption in diets of monogastric animals (pigs) and ruminants (cattle). FA: Fat; DAG: Diacylglycerol; FFA: Free fatty acids; MG: Monoglyceride; TBA: Total bile acid; LCFA: Long chain fatty acid; MCFA: Medium chain fatty acid; SCFA: Short chain fatty acid; APO: Apolipoprotein; TAG: Triacylglycerol; CD36: Cluster of differentiation 36; FABP: Fatty acid-binding protein; FATP: Fatty acid transport protein

As shown in Fig. 2, the first step in the digestion of fat in diet occurs in the stomach; this process is catalyzed by lingual lipase or gastric lipase [24]. Specifically, lingual lipase is predominant in ruminants and rodents, whereas gastric lipase is predominant in swine, rabbits, dogs and humans [25]. The stomach digests about 10%–30% of triglycerides in diet, and the resulting products are diglyceride and free fatty acids [24]. The remaining undigested lipids are discharged as an emulsion encapsulated by cholesterol and cholesterol esters from the stomach. Although gastric lipase catalyzes limited fat digestion, it aids pancreatic lipase in binding to the emulsion/water interface, allowing fat digestion to be more efficient [26]. Pancreatic cholesteryl ester hydrolase can fully hydrolyze cholesteryl esters into free fatty acids and free cholesterol [27], and it may also have a role in the digestion of triglyceride containing LCPUFAs [27]. Phospholipids in feed are degraded to 1-lysophospholipids and free fatty acids by activated pancreatic phospholipase A2 [27]. Ultimately, the intestine absorbs all fat digestion products into circulation.

The ability and mechanism of fatty acid absorption are first determined by the carbon chain length. Fats containing fatty acids with less than 8 carbon atoms can be absorbed without hydrolysis, but those containing fatty acids with more than 8 carbon atoms must be absorbed after enzymatic hydrolysis, and short- (≤ 4 carbons) and medium-chain (≤ 12 carbons) fatty acids are more easily absorbed by the intestine than long-chain fatty acids [28]. Short-chain fatty acids are currently thought to enter the gut in two ways. First, short-chain fatty acids pass through the cell *via* simple diffusion, while formic acid, ethanoic acid and propionic acid can enter the cells *via* nonionic diffusion and penetration, similar to other electrolytes in the lipid bilayer [29]. Second, short-chain fatty acid transport requires certain proteins, and the entry of butyric acid into the rat distal colon is defined as pH-dependent and carrier-mediated anion exchange [30, 31]. Moreover, fatty acid transport proteins are not required for the intake of short-chain fatty acids in the gut [32]. Intestinal cells absorb medium-chain fatty acids with more than 8 carbon atoms in a similar way with long-chain (> 12 carbons) fatty acids. The main difference is that the rate of long-chain fatty acids passing through the unstirred water layer (a liquid membranes on the surface of the epithelium) is limited and that of medium-chain fatty acids is limited only on the brush border membrane [33]. Fatty acid transport proteins [32], FAT/CD36 [34] and fatty acid-binding protein (FABP) [35] are required for long-chain fatty acid uptake.

Functional fatty acids are used by livestock in a variety of ways during the reproductive cycle; with 3 primary functions. (1) After digestion and absorption, fatty acids are induced to form lipid droplets in cells. Different types of fatty acids are stored by different forms in the tissues and cells of livestock to exert key biological function. Except for the liver and adipose tissue, most tissues are strongly reliant on fatty acids. However, large numbers of lipid droplets are found in livestock oocytes and embryos [36, 37]. Such a high-density lipid accumulation condition is linked to their vital role in reproduction, and the lipid composition of lipid droplets can be regulated by lipids in diet [38]. (2) Fatty acids in vivo, whether endogenously synthesized or derived from diet, can be utilized by an organism to provide cellular energy *via* mitochondrial β-oxidation. The full oxidation of fatty acids can yield 38 kJ/g energy, while only 17 kJ/g energy can be produced by carbohydrates and proteins. Whereas oocytes require a substantial amount energy during development, especially during cytoplasmic and nuclear maturation, and frequent cell division and differentiation during embryo development also depend on an abundant energy supply. Such an efficient energy supply plays a crucial role in oocyte maturation and embryo development in livestock [14]. (3) In addition, fatty acids are metabolized into other functional molecules, such as cannabinoids, PGs, lysophospholipids, sphingosine 1-phosphate and steroid hormones, that are important for regulating livestock reproduction [10]. Therefore, altering the fatty acid structure in diet can not only supply energy to livestock oocyte and embryo development but also improve the lipid environment for oocyte and embryo development.

### Effects of functional fatty acids on follicular development in livestock

Over the years, researchers have discovered a strong link between oocyte quality and follicle size [39–41]. Folliculogenesis involves paracrine, autocrine and endocrine interactions, which are dependent on the proliferation and differentiation of granulosa cells and nutrients in the follicular fluid (FF), thereby jointly creating an important and unique microenvironment for oocyte development and maturation. The follicle in mammals is a functional syncytium including the oocyte, the cumulus cells, and the mural granulosa cells [42]. And the granulosa cells (corona radiata) which are directly attached to the zona pellucida extend membrane transzonal projections that form gap junctions with the vitelline membrane of the developing oocyte [43]. Therefore, follicular growth and oocyte maturation are associated with dynamic transcriptional regulation of oocytes and granulosa cells [44]. Follicular fluid, a key dynamic component of the follicle, reflects the follicular developmental status and is an indicator of granulosa and theca cell activity [45]. According to recent findings, functional fatty acids improve follicular growth and consequently oocyte quality in livestock by influencing steroidogenesis in granulosa cells/corpus luteum as well as the lipid composition of FF.

### Improvement of the steroidogenesis capacity in granulosa cells/corpus luteum through functional fatty acids to promote follicular development in livestock

Although a variety of cells in the follicle regulates steroidogenesis, only the effect of fatty acids on granulosa cell function has been widely reported, especially in livestock. Depending on stage of development and location of the granulosa cells within the follicle wall and surrounding the oocyte, granulosa cells have distinct phenotypes (mural granulosa cells, sinus granulosa cells, and cumulus granulosa cells). Mural granulosa cells have a great capacity for steroidogenesis [46]. Estradiol is the important steroid hormone produced by granulosa cells before ovulation, whereas cumulus granulosa cells, which are discharged with the oocyte at ovulation, do not express aromatase but can secrete extracellular matrix components such as hyaluronic acids proteoglycans [47, 48] which are involved with expansion of the cumulus cells during ovulation. Granulosa and theca cells become luteal cells and are responsible for the production of estradiol and progesterone, the latter is predominantly expressed in the corpus luteum [49–51]. The ability of granulosa cells/corpus luteum to synthesize steroid hormones is the most important function.

**Fig. 3:**
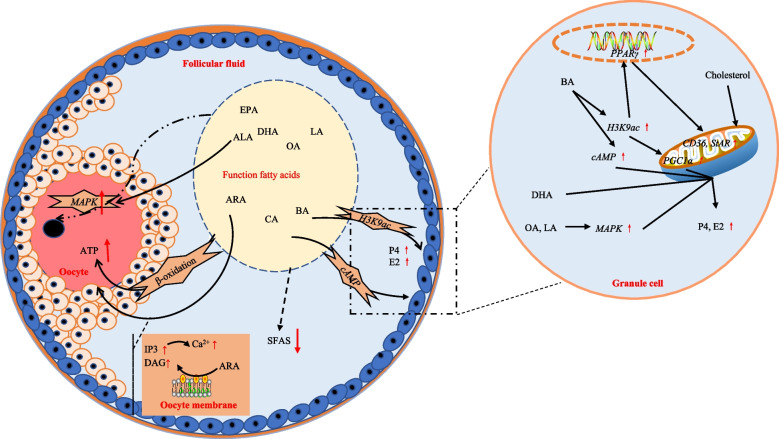
Molecular mechanisms of functional fatty acids affecting follicular development, granulosa cell steroidogenesis, and oocyte maturation. PPARγ: Peroxisome proliferator-activated receptor gamma; PGC1α: Peroxisome proliferator-activated receptor gamma coactivator 1-alpha-like; CD36: Cluster of differentiation 36; StAR: Steroidogenic acute regulatory protein; BA: Butyric acid; H3K9ac: Acetylation of histone 3 at lysine 9; cAMP: Cyclic Adenosine monophosphate; DHA: Docosahexaenoic acid; OA: Oleic acid; LA: Linoleic acid ; MAPK: Mitogen-activated protein kinase; P4: Progesterone; E2: Estradiol; ALA: α-linolenic acid; EPA: Eicosadienoic acid; ATP: Adenosine triphosphate; IP3: Inositol 1,4,5-triphosphate; DAG: Diacylglycerol; ARA: arachidonic acid; SFAs: Saturated fatty acid; CA: Caprylic acid; ↑: Promote/Increase; ↓: Inhibit/Decrease.

Research on the effects of fatty acids on granulosa cells has been widely conducted over the last two decades because fatty acids, particularly long-chain unsaturated fatty acids, are considered precursors for steroidogenesis. Both etomoxir, which inhibits fatty acid oxidation, and C75, which inhibits fatty acid synthesis, have been found to impair DNA synthesis in bovine granulosa cells and reduce the phosphorylation of adenosine monophosphate-activated protein kinase (AMPK) and acetyl-CoA carboxylase [52]. Furthermore, C75 inhibits the synthesis of progesterone in granulosa cells [52]. These findings imply that fatty acid oxidation and synthesis are critical for granulosa cell proliferation and steroidogenesis. Previous in vitro culture studies revealed that OA (oleic acid) can boost estradiol synthesis in bovine follicular granulosa cells cultured in vitro but reduce the proliferation of granulosa cells [53]. However, in recent research, OA is reported to have adverse impacts on the steroidogenesis and morphology of bovine granulosa cells cultured in vitro [54]. Similarly, in vitro culture experiment involving bovine follicular granulosa cells revealed that α-linolenic acid (ALA) and LA inhibit the expression of genes associated with steroidogenesis in granulosa cells and lower the concentrations of secreted estradiol and progesterone [55]. But, the treatment of bovine granulosa cells with another functional fatty acid, DHA (docosahexaenoic acid), promoted granulosa cell proliferation as well as progesterone and estradiol secretion [56]. A low-dose ARA (arachidonic acid) boosted the survival rate of granulosa cells, but high-dose ARA suppressed estradiol secretion and promoted progesterone synthesis in granulosa cells [57]. The differences suggest that both the concentration and type of fatty acid have an effect on steroidogenesis. Although functional fatty acids have been found to affect steroid synthesis, the specific regulatory mechanisms remain unclear. It has been found in goats that OA and LA boost the secretion of progesterone from goat granulosa cells through the mitogen activated protein kinase (MAPK) ERK1/2 signaling pathway [58]. Butyric acid is found to stimulate the synthesis of progesterone and estradiol in porcine ovarian granulosa cells *via* the cAMP signaling pathway [59]. Also, our previous research showed that butyrate triggers histone acetylation of histone H3K9 (H3K9ac) to activate steroidogenesis through peroxisome proliferator-activated receptor gamma (PPARγ) and peroxisome proliferator-activated receptor gamma coactivator 1-alpha-like (PGC1α) pathways in ovarian granulosa cells [60] (Fig. 3). In addition, there are few studies on combinations of functional fatty acids, but several in vivo feeding trials have found that a supplementation of LCPUFAs can promote ovarian steroidogenesis, which suggests targeted use of specific fatty acid combinations in physiological stages may have positive CL effects on granulosa cell function [61–65].

More studied are required in terms of the impact of fatty acids, especially functional fatty acids, on the function of ovarian granulosa cells/corpus luteum in livestock, not only in terms of steroid hormone, but also in terms of the effects of fatty acids on follicle stimulating hormone (FSH) and luteinizing hormone (LH), and more combinations should be explored. In vivo experiments combined with specific physiological stages of livestock may be more effective in clarifying the role of functional fatty acids in granulosa cells/corpus luteum.

### Enhancement of follicular development in livestock by improving the composition of functional fatty acids in FF

The only environment for oocyte growth and maturation is FF, while FF comes from 2 sources: blood flow connecting some sheathed capillaries in the ovary cortex and components secreted by the cellular layer within the follicle, especially granulosa cells [45, 66]. FF is rich in fatty acids, with nonesterified fatty acids (NEFAs) being the most prevalent, and the content and concentration of NEFAs are affected by maternal physiological circumstances [13, 67]. LA, OA, stearic acid, palmitic acid, AA and ALA are the primary components of FF lipids from swine, cattle and goats (Table 2), indicating that functional fatty acids are significant component of FF, and further investigation of their role and changes in composition and concentration are important. Furthermore, a recent metabolomics analysis of FF in high- and low-reproduction sows indicated that the fatty acid composition and metabolism of sows with varying reproductive performance differ significantly [68].

**Table 2:** Fatty acid composition of follicular fluid from different livestock

Fatty acid, %	Livestock		
	**Prcine** [69]	**Cow** [70]	**Goat** [71]
C16:0 (palmitic)	24.73	26.6–30.3	24.59
C18:0 (stearic)	15.5	22.8–28.8	22.66
C18:1 (oleic)	17.81	14.6–19.3	23.53
C18:2 (linoleic)	16.77	23.6–30.8	10.01
C18:3 (α-linolenic)	0.95	-	2.22
C20:3 (homogamma linolenic)	-	0.7–1.3	-
C20:4 (arachidonic)	10.8	1.8–3.5	5.92
C22:5 (eicosapentaenoic)	0.12	-	1.89
C22:6 (docosahexaenoic)	0.85	-	1.91

Lipids taken up by oocytes are mainly from FF. Changes in the lipid environment of FF have a strong impact on oocyte maturation. Lipid-rich FF induces lipid accumulation and endoplasmic reticulum stress in oocytes, weakening oocyte maturation [72], and such changes affect early embryo development and subsequent pregnancy [73–75]. Therefore, the kinds of fatty acids should be considered at the time of improve the lipid environment of FF in livestock. Continuous feeding of fatty acid calcium soap to cows for 150 d after parturition altered the fatty acid composition in follicles and greatly increased the proportion of estradiol-rich active follicles and large follicles [76]. Another study involving the follicular injection of fatty acids showed that OA reduced considerably reduced the concentration of estradiol in follicles and the follicular ovulation rate [77]. Fatty acids are influenced by the body’s complex physiological environment; therefore, they may transform or affect follicular development in other ways. According to comparative research, the FF of dominant follicles of cattle have large quantities of ALA and low concentrations of OA and ARA [70]. Consistent results with goats were obtained in a comparative analysis of goat follicles, i.e., the concentration of ALA in FF rises with the enlargement of follicles [78]. As demonstrated in horse follicle culture experiments, ALA added into the medium can facilitate horse follicular growth and granulosa cell proliferation [79]. Using in vivo experiments, researchers also discovered that adding linseed oil to cattle diet can increase the proportion of ALA, EPA and DHA in FF and granulosa cells, contributing to oocyte maturation and embryo development [80]. Likewise, ALA-rich sow diet can greatly improve the composition of these fatty acids in FF and improve oocyte quality [69]. These findings imply that an increase in ALA concentration in FF has a positive influence on livestock follicular growth despite an unclear mechanism. In sum, the lipid composition of FF, i.e., the lipid environment in which the oocyte is located, is well understood, but there has been little research on the functions and changes in key fatty acids, especially on the molecular mechanisms of lipid regulation throughout follicular development. Further clarifying the exact functions and mechanisms of lipids during livestock follicular development will help increase oocyte quality and assure pregnancy success.

## Role and mechanism of functional fatty acids in oocyte maturation and development in livestock

The oocyte is the largest cell in terms of diameter in female livestock, and as the female germ cell in sexual reproduction, its normal and high-quality development is the foundation for animal reproduction. Before oocyte fertilization into early embryo and embryo implantation, the oocyte provides practically all of the nutrients and associated substrates for the fertilized egg. Therefore, it is of great importance to study the effects of different nutrients on oocyte quality. However, most research has focused on oocyte metabolism of exogenous nutrients such as glucose, lactic acid, pyruvic acid and amino acids, while lipid metabolism has been largely ignored. As previously stated, livestock oocytes have significant lipid droplet enrichment, and the analysis of fatty acids in porcine oocytes revealed that palmitic acid is the most abundant in total and neutral lipids, followed by OA, while n-6 PUFAs, such as LA, ARA and adrenic acid, also account for a large proportion [81]. Triglycerides are the most abundant in porcine oocytes (74 ng each oocyte), 3 times that in bovine and goat oocytes [82]. The total fatty acid content is also high in porcine oocytes (160 ng each oocyte), 2.5 times that in bovine oocytes and 1.8 times that in goat oocytes. Palmitic acid, stearic acid and OA are the most abundant in bovine, porcine and goat oocytes, respectively, but swine has higher palmitic acid content than OA content, while cattle and goats have higher relative OA content. Although oocyte size is not taken into account, Nile red staining revealed that the content of lipids in porcine oocytes is 2.4 times that in bovine oocytes, while that in bovine oocytes is 2.8 times that in mouse oocytes [83].

### Role of fatty acid oxidative energy supply in oocyte development and maturation

The primary function of fatty acids in oocytes is to provide energy. Meiosis in mammalian oocytes is arrested as early as the embryonic period in the diplonema of the first meiosis prophase, and oocytes cannot resume meiosis until exposure to gonadotropins following sexual maturity. Failure of resumption of oocyte meiosis leads to maturation failure, significantly decreasing livestock reproductive performance. Oocytes require a substantial amount of energy during meiotic resumption. Fatty acids can be used as the sole source of energy for oocyte growth and development, as in vitro oocytes in dairy cows have been shown to mature successfully in the absence of external energy sources [84]. Oocyte maturation in vitro is delayed when fatty acid β-oxidation is blocked and an external energy source is unavailable [85]. The use of methyl palmitate (a targeted inactivator of carnitine palmitoyl transferase 1, to block the entry of fatty acids into the mitochondria [86]) to inhibit fatty acid metabolism during the in vitro maturation (IVM) of porcine and bovine oocytes has been shown to reduce oocyte viability and the blastocyst rate after oocyte fertilization [85, 87]. Similarly, acyl-CoA synthetase long-chain family member 3 (ACSL3) and acyl-CoA synthetase long-chain acyl-CoA dehydrogenase (ACADL) are activated before long-chain fatty acids enter the mitochondria and catalyze the first step of β-oxidation, and the levels of these two enzymes are dysregulated in porcine oocytes with limited developmental capacity [88]. Furthermore, the in vitro rate of mouse mature cumulus-oocyte complexes (COCs) to metabolize fatty acids is less than half that of mature COCs in vivo, and the rate is linked to dysregulated expression of at least 15 genes involved in fatty acid activation, transport, and oxidation [89]. All of the above findings suggest that fatty acid oxidative energy supply is critical during oocyte growth and meiotic maturation but that the elevated levels of different fatty acids have different significance for oocytes based on their types and structures. Excessive levels of fatty acids, especially saturated fatty acids, in oocytes are now widely known to cause lower fertilization and embryo development rates as well as irreversible damage to fetal growth and offspring [90, 91]. This is linked to oocyte mitochondrial morphological damage and oxidative stress induced by excess fatty acids [92]. OA can counteract this unfavorable effect by assisting in the absorption and stable storage of these saturated fatty acids in lipid droplets [37]. Further studies are needed on the role of different fatty acids in oocyte β oxidation. A more balanced functional fatty acid composition in oocytes not only provides enough energy but also minimizes lipid-related unfavorable consequences.

### Other roles of functional fatty acids in oocyte development and maturation

Fatty acids can influence oocyte maturation in a variety of ways in addition to the β-oxidation pathway. When ALA is added to bovine oocytes during IVM, the number of cells reaching meiosis II increased, and the MAPK signaling pathway is more active in these oocytes, resulting in higher-quality embryos [93]. A supplementation of 0.1 µmol/L of DHA significantly improved oocyte growth, increased acetylation levels of H4K12, and ATP contents [94]; LA inhibits the maturation of bovine oocytes in vitro [95], suppresses embryo division and blastocyst development, and weakens the MAPK1/3 signaling pathway [96]. Furthermore, phospholipid and cholesterol enrichment during oocyte maturation is required for rapid cell division and membrane formation after oocyte fertilization. During oocyte maturation and embryo development, phospholipids are also involved in the synthesis of second messengers. Phosphatidylinositol, for example, makes up 6% of total phospholipids in porcine oocytes and is rich in ARA and stearic acids as well as palmitic acid [81]. Inositol 1,4,5-triphosphate (IP3) and diacylglycerol (DAG) are two second messengers produced by membrane phospholipid hydrolysis. IP3 and its derivative (IP4) increase Ca^2+^ levels, whereas DAG stimulates PKC [82, 97], both of which are critical in oocyte maturation and single-sperm fertilization capacity. There are many other functional lipids in oocytes similar to phospholipids, but it is still unknown how they, including phospholipids, are affected by fatty acid levels in ration and the body. It is clear that the correlation between lipid oxidation in oocytes and oocyte maturation has been well explored. However, the regulation of fatty acids on signaling molecules in oocytes and the role of membrane structure have rarely been studied; further research is needed.

## Mechanism of functional fatty acids in increasing the livestock implantation rate by improving endometrial receptivity

Embryo implantation is the most critical step in a successful pregnancy, which involves the regulation of implantation, embryo development, uterine physiology and the interaction between embryo and uterus. Receptivity refers to the endometrium’s ability to allow for normal implantation, and optimal receptivity ensures normal implantation, which is the foundation for a healthy pregnancy [98]. The majority of embryo losses in livestock occur during the implantation period, and embryo implantation failure in early pregnancy is a leading cause of pregnancy failure. Embryonic loss in pigs, for example, is the highest among livestock, ranging from 30% to 50% [99]. Embryo loss in the early pregnancy accounts for about 75% of the entire pregnancy [100]. Multiple reaction processes, such as immune responses, inflammatory responses, complement pathway changes and coagulation modulation, are regulated by the transcriptomic profiles of the receptive endometrium [101]. Triglycerides and eicosanoids are the major lipid mediators released by the endometrium during the implantation period. In the eicosanoid family, PGs, thromboxanes, leukotrienes, endogenous cannabinoids and sphingolipids all play roles in reproduction [102–104]. In addition, steroid hormones and lysophospholipids produced under direct or indirect regulation of lipid metabolism are also important regulators of endometrial receptivity.

### Functional fatty acids regulate steroidogenesis and improve endometrial receptivity

The endometrium is extremely sensitive to hormonal changes, especially in the presence of steroid hormones, and such changes help the embryo prepare for implantation [105]. Estrogen and progesterone are key mediators of embryo implantation. During this critical period, estrogen and progesterone are still supplied by the ovaries. In both in vivo and in vitro experiments, functional fatty acids have demonstrated to influence estrogen and progesterone levels in livestock [106–108]. Estrogen, in particular, plays a critical role in the embryo implantation window, and high estrogen levels can cause the window to close in some mammals [109]. Estrogen and progesterone primarily act through nuclear receptors, namely estrogen receptor (ER) and progesterone receptor (PR). Estrogen binding to ER is primarily responsible for endometrial epithelial cell proliferation [110]. Nuclear receptor coactivator-6 (NCOA6) degrades ER by ubiquitination, while NCOA6 loss in the uterus disrupts embryo implantation by increasing E2 sensitivity [111]. it is found that initiation of progesterone receptors (PRs) in the uterus and uterine epithelium by progesterone 10–12 days after estrus is essential for achieving receptivity of the endometrium for implantation in pigs [112]. Furthermore, PR is dependent on steroid receptor coactivator 2 (SRC2) to activate the uterine decidual reaction; therefore, *in utero* knockout of SRC2 results in implantation failure [113, 114]. Therefore, these studies indicate that fatty acids regulate the receptivity of endometrium in early pregnancy by mediating the levels of estrogen and progesterone.

### Functional fatty acids improve endometrial receptivity by affecting the synthesis of PGs

Enhanced vascular permeability at the blastocyst implantation site is associated with increased endometrial receptivity in livestock [115]. PGs have long been recognized as key vascular active factors in ovulation, fertilization, late pregnancy and delivery. PGs have also been discovered to be essential for the success of embryo implantation [102, 116]. PGs are lipid mediators generated by the enzymatic metabolism of ARA, a 20-carbon unsaturated fatty acid. In response to numerous physiological and pathological stimuli, ARA is produced *via* cell membrane phospholipids under the catalysis of phospholipase A_2_ (PLA_2_) and converted into the PG intermediate metabolites PGG_2_ and PGH_2_ in turn under the epoxidation and peroxidation activity of prostaglandin H synthase (PGHS), also known as prostaglandin-endoperoxide synthase (PTGS). After being metabolized by different downstream PG synthases, various biologically active PGs are generated, including PGI_2_, PGE_2_, PGF_2__α_, PGD_2_, and thromboxane A2 (TxA2). PTGS is a major enzyme in PG synthesis, with 2 isoforms, PTGS-1 and PTGS-2, found in the endoplasmic reticulum and nuclear membrane in the form of homodimers or heterodimers. PTGS-1 and PTGS-2 are functionally different but interrelated, and both are involved in maintaining the homeostasis and synthesis of PGs during inflammation. PGE_2_ maintains the luteal function for embryo development and early implantation [117]. PGF_2__α_ is a main luteolytic factor in vivo. In sows, PGF_2__α_ is an important regulator of corpus luteum function, uterine contractility, ovulation, and embryo attachment [118]. Also, the regression of the corpus luteum in ruminants is initiated by the rhythmic release of PGF_2__α_ in high concentrations from the non-pregnant uterus [119]. Functional fatty acids in diet have been shown to influence PG synthesis in a variety of ways, including the provision of substrates in PG synthesis, influencing the expression and concentration of associated enzymes, and acting as substrates and competitive inhibitors of cyclooxygenases [120]. Furthermore, the proportion of functional fatty acids, especially PUFAs, in diet alters the phospholipid composition in the cell membrane, indicating the importance of fatty acids, because different PG precursors compete for the same enzyme system [121]. Previous research has demonstrated that adding functional fatty acids to feed can regulate the gene expression of important enzymes in the PG biosynthesis pathway, such as PTGS [122], in the first trimester of pregnancy, thereby benefiting the overall porcine reproductive process [123].

In addition to the above 2 major lipid metabolic pathways that alter endometrial receptivity in livestock, lysophospholipids [124], endogenous cannabinoids [125] and sphingolipids [10] are also regulated by lipid metabolism and play crucial roles in endometrial receptivity. But there is little direct evidence to confirm that functional fatty acids are involved in regulating their synthesis and function, further research is needed.

## Role of functional fatty acids in placental vascular development

In livestock, the placenta supports normal embryo development and growth during pregnancy. The most important function of the placenta is to carry nutrients, gases, and waste between the maternal and fetal circulations, thereby creating a favorable environment for embryo development in the uterus [126]. The maternal and fetal circulations are separated by multiple layers of cells; therefore, factors at the maternal-fetal interface regulate the transfer of molecules between these layers [127]. IUGR and miscarriage are two examples of embryonic defects caused by poor placental function [126, 128]. Placental malnutrition has a long-term impact on offspring metabolism, even throughout life [129]. Fatty acids are transported from the mother to the child through the placenta and, together with their metabolites, are also stored in considerable amounts in the placenta, supporting placental growth and development as the pregnancy proceeds. Based on recent research, fatty acids are thought to influence placental growth and development through regulating placental angiogenesis.

The development of the placental vascular network is vital to the growth and maintenance of the developing embryo [130–132]. Vascular endothelial growth factor (VEGF), angiopoietin-like protein 4 (ANGPTL4), platelet-derived growth factor (PDGF) and platelet-activating factor (PAF) are all involved in the process of angiogenesis [130–133]. Angiogenesis can be aided by functional fatty acids either directly or indirectly [134–136]. Studies have shown that angiogenic growth factors, cell migration, proliferation and angiogenesis are all regulated by eicosanoid, and eicosanoid produced by ARA boosts angiogenesis, while eicosanoid produced by EPA and DHA suppresses angiogenesis [134–136]. Angiogenesis is influenced by a variety of different elements [135, 137]. Prostaglandin E_2_ (PGE_2_) is also implicated in placental angiogenesis [135, 138, 139], and it boosts the production of VEGF, bFGF and CXCL1, which in turn promote angiogenesis through targeting endothelial cells [135, 139–141]. Furthermore, PGE_2_ is a PTGS-2 product, and there is considerable evidence that PTGS-2 is an angiogenic mediator; the inhibition of PTGS-2 with selective PTGS-2 inhibitors can significantly prevent inflammation, proliferation and angiogenesis and induce apoptosis [142]. A range of angiogenic factors, including VEGF, ANGPTL4, PDGF, leptin and TNF, have been shown to be regulated by functional fatty acids and their derivatives [135, 136]. N-3 functional fatty acids influence angiogenesis through a variety of mechanisms, including regulating the expression of VEGF, ANGPTL4 and other mediators such as eicosanoid, PTGS, FABP and nitric oxide (NO) [135]. In human placenta, the extra villous trophoblast cells, DHA stimulates angiogenesis by boosting the expression and secretion of VEGFA, the most potent angiogenic factor [143]. Therefore, DHA can help early placental development by boosting angiogenesis [143]. The mechanism by which DHA increases VEGFA expression in placental trophoblast cells remains unknown. However, DHA is specific for VEGFA expression, because it’s mRNA is induced by a wide range of growth factors and cytokines, including PDGF, EGF, TNF, TGF-1 and IL-1, not by other fatty acids [143]. In these cells, DHA-induced VEGFA expression is not accompanied by PTGS-2 and HIF1 (Hypoxia inducible factor 1) expression, suggesting that DHA metabolites may not be involved in VEGFA expression. The DHA stimulation of VEGFA expression is unlikely to involve PPARγ because PPARγ ligands do not promote VEGFA expression in these cells [144]. DHA enhances VEGFA expression and secretion, whereas fatty acids, including EPA, ARA, OA and CLA, promote ANGPTL4 secretion without influencing VEGF synthesis in placental trophoblast cells. These findings imply that the mode of action of DHA in angiogenesis is distinct from other fatty acids [143]. The mechanism by which fatty acids other than DHA stimulate ANGPTL4 secretion in placental trophoblast cells is still unknown. Based on recent extensive research on angiogenesis regulators in human and mouse, functional fatty acid regulation of placental angiogenesis may also become a unique and effective way to ensure a favorable outcome in most pregnancies of livestock. Of course, more relative researches on livestock are needed in the near future.

## Role of functional fatty acids in livestock embryo development

Reduced fertility is generally caused by inferior oocyte and embryo quality rather than ovarian/endocrine dysfunction [145–147]. Moreover, enhancing livestock reproductive performance by assisted reproduction techniques, such as in vitro fertilization and embryo transfer, has relied on high-quality early embryos after in vitro fertilization. Despite differences in gestation period and number of fetuses across livestock species, the embryo development stage is essentially the same. From embryogenesis to delivery, there are 4 major morphological changes: fertilized egg, morula, blastocyst and fetus. Fatty acids play vital roles in each stage of embryo development by supplying energy, controlling steroid hormones, and serving as signaling molecules and membrane structures. Even though the fetus has a partial capacity to endogenously synthesize fatty acids [148–150], the embryo is still primarily supplied with fatty acids by the mother throughout embryo development, and the corresponding requirements for fatty acids also vary as the embryo development stage changes, all of which have been confirmed in studies involving humans and livestock over the last 3 decades [151–156].

### Role of functional fatty acids in early embryo development

The development from fertilized egg to blastocyst is a continuous and short process compared to the entire gestation period, during which any developmental disorder can have a negative impact on the pregnancy outcome. Fatty acids are important not only for the storage of energy substrates but also for the maintenance of membranes, which is of significance because of the substantial increase in the plasma membrane surface area during embryo division. The plasma membrane surface area increases by 74% between the 2- and 4-cell stages, implying an even larger increase in the late preimplantation period [157]. The fat content in the embryo decreases dramatically as it develops [158]. The embryo has a higher predilection for n-3 functional fatty acids at this stage, which has been well evidenced. When compared to diet rich in palmitic acids and stearic acids, diet rich in ALA and LA can improve bovine blastocyst quality [159]. However, adding LA to bovine oocytes cultured in vitro reduces the oocyte cleavage and blastocyst rates, and further mechanistic research has revealed that LA inhibits the phosphorylation of the AKT and MAPK1/3 signaling pathways during oocyte maturation [160]. In addition, the results from mouse studies also indicate that LA decreases the development rate of fertilized eggs at the 1-cell and 2-cell stages and that LA produces a greater proportion of oxygen radicals, thus inducing oxidative stress [161]. Diet with a high n-3/n-6 ratio boosts ALA and estradiol levels in bovine follicles and improve the embryo cleavage rate [162], while conjugated LA reduces the embryo development rate and hinders the expression of stearoyl-CoA desaturase-1 (a synthetase transforming stearic acid into OA) [163]. Another study involving pigs compared DHA and EPA and showed that adding DHA to IVM medium may benefit porcine oocyte development, whereas EPA displays cytotoxicity [164]. As demonstrated in a study on the influence of OA and palmitic acid on the mouse embryo development in vitro, the combined use of the two acids had a positive effect on blastocyst formation, whereas OA alone is superior to palmitic acid [165]. Similarly, a recent study on pigs found that 150 µmol/L OA can enhance the blastocyst rate of parthenogenetically activated porcine embryos [166]. Individual fatty acids have a positive effect on embryo development, but an appropriate combination of fatty acids appears to be more adapted to real physiological conditions and may have more specific effects. In studies examining the effects of palmitic acid, OA, ALA and ARA alone or in combination on 8-cell rat embryo development in vitro, OA, ALA and ARA improved the development from 8-cell embryos to blastocysts. This is especially true when carbohydrates were not present. OA is the most efficient among them, and the addition of palmitic acid did not improve embryo development with or without carbohydrate substrates. Addition of the mixture of four fatty acids is more effective for rat embryo development than single treatment with any of fatty acids tested [167]. The research on fatty acids have greatly improved with the maturation of biotechnology and improvements in chemical synthesis techniques, but there is still a lack of understanding of the precise fatty acid nutrition in livestock embryos, the types of fatty acids in diet are often limited, and studies on single or combined lipid use are severely lacking.

### Role of functional fatty acids in embryo development in the second and third trimesters of pregnancy

As pregnancy proceeds, the embryo gradually develops tissues and organs after the blastocyst stage, during which fatty acid deficiency and maternal lipid metabolism disorders can easily result in fetal growth restriction, malnutrition, preterm delivery, and even miscarriage [168]. At this time, fatty acids play a positive role in hormone maintenance and energy metabolism, and active maternal fatty acid transport is required for maintaining fetal development. DHA and ARA are considered to be the most important structural components of the fetal central nervous system, and they are transferred through the placenta and accumulate in the brain and other organs during fetal development [169, 170]. Furthermore, recent research has revealed that medium-chain fatty acids also play a vital role in maternal-fetal metabolism [171–174]. Medium-chain fatty acids are an ideal substrate for mitochondrial energy production, especially for fetuses because fetuses have a high requirement for energy due to the inefficiency of their enzyme system. Among them, caprylic acid, which is abundant in cord blood, is vital for the newborn energy supply [174]. Researchers have shown that lauric acid (C12:0) may affect PUFA in animal models [175] and that it may be a precursor to the n-3 LCPUFAs in the fetus under some conditions. In contrast to early gestation research, a substantial number of in vivo trials have been undertaken with regard to late gestation; the effects of several types of functional fatty acids added to livestock diet on reproductive performance are summarized in Table 3. As shown in the table, adding various functional fatty acids in late gestation can positively increase livestock reproductive performance and alter reproductive performance through hormone synthesis, antioxidative stress and energy supply.

**Table 3:** Effects of dietary functional fatty acids on reproductive performance of livestock

Livestock	Oil source	Stage of pregnancy	Main findings and potential mechanism	Reference
Sows	Butyrate	Late pregnancy	Shorten the weaning-to-estrus interval	[176]
Sows	Butyrate	Pregnancy and lactation	Reduced the rate of gilts return to estrus, alter the composition of colostrum and enhance the growth rate of piglets	[177]
Ewes	Oleic	Late gestation	Increased PGE_2_ production in both endometrium and fetal allantochorion cells and increasing the ratio of PGE_2_ to PGF_2α_ in endometrium cells	[178]
Sows	Conjugated linoleic acid	Late pregnancy and lactation	Reduced backfat thickness loss during the lactation period and leading to higher piglet weight at weaning	[179]
Ewes	Linoleic acid	Late pregnancy	Enhanced placental PG production by increased the supply of 20:4n-6	[180]
Cows	Conjugated linoleic acid	Period started 21 d pre-calving and continued until 60 d in milk	Increased conception rate and serum concentrations of glucose, cholesterol, triglyceride (TG), insulin, insulin-like growth factor-1(IGF-1), estradiol and progesterone were higher	[181]
Cows	α-linolenic acid	55 ± 22 d postpartum	Increased the size of the ovulatory follicle and reduced pregnancy losses	[182]
Cows	α-linolenic acid	21 d before expected calving	Decreased incidence of ketosis and severe metritis, reduced mortality, and tending to enhance fertility performance	[183]
Sows	Linoleic acid and α-linolenic acid	Pregnancy	Rapid return to estrus, increased maintenance of pregnancy and improved subsequent litter size	[184]
Cows	DHA	From 27 to 147 d postpartum	Enhanced embryo development based on changes in interferon-stimulated gene expression	[185]
Sows	Fish oil	60 d before parturition to weaning	Increased subsequent litter and litter size	[19]
Sows	Marine algae	Five days prior parturition to breeding	Increased subsequent litter and litter size	[186]

## Role of functional fatty acids in livestock during the lactation period

The lactation period is important for the rapid growth and development of young livestock and crucial for the transition of maternal livestock to the next reproductive cycle. Maternal nutritional needs during the lactation period are determined by diet supplements and maternal body reserves. Maternal nutritional imbalances during the lactation period often cause malnutrition, growth retardation and even death in young livestock as well as a greater loss in maternal body reserves, which then impairs subsequent estrus and pregnancy and even shortens the durable years of maternal livestock [187, 188]. Fatty acids can reduce weight loss of maternal livestock and improve the hormones level for estrus and the ovarian and uterine microenvironment during mating while providing enough energy to maintain a high milk yield. Therefore, fatty acid nutrition is highly important during the lactation period of maternal livestock. The lipid composition of milk is largely identical but with minor differences among different species. Table 4 summarizes the composition of fatty acids in livestock milk [189–191]. In livestock production, changing the fatty acid composition of diet has been found to impact the lipid composition of milk and the milk yield [192–198]. Adding functional fatty acids to the diet of maternal livestock can influence the fatty acid composition of young livestock, which is a desired outcome [199, 200], and help boost neurodevelopment, immunology and intestinal protection [60, 201–205]. Fatty acids in sows and dairy cows during the lactation period have been extensively studied recently.

**Table 4:** Composition of fatty acids in milk of different livestock

Fatty acid, %	Livestock		
	**Prcine** [189]	**Cow** [190]	**Goat** [191]
C4:0 (butyric) xinsuan	-	2.95	1.37
C8:0 (caprylic)	-	1.18	6.17
C12:0 (dodecanoic)	0.27	3.06	6.20
C16:0 (palmitic)	31.04	30.7	21.58
C18:0 (stearic)	4.38	9.12	8.58
C18:1 (oleic)	30.26	21.2	16.56
C18:2 (linoleic)	18.17	3.59	0.99
C18:3 (α-linolenic)	1.14	0.5	0.66
C20:3 (homogamma linolenic)	0.08	-	0.26
C20:4 (arachidonic)	0.44	-	0.05
C22:5 (eicosapentaenoic)	0.10	-	0.02
C22:6 (docosahexaenoic)	0.19	-	-

### Role of functional fatty acids in sows during the lactation period

In actual sow reproduction, diet is dominated by grain and protein feeds and contains very little n-3 LCPUFAs. In recent years, the optimal n-6:n-3 ratio during the lactation period has been a research hotspot. It is now thought that the optimal n-6:n-3 ratio is about 9:1 or 10:1 [206, 207]. Based on this, there has been substantial interest in the last decade in determining whether adding n-3 functional fatty acids affects the health of sows and piglets during the lactation period. According to recent research, n-3 functional fatty acids can lower mortality [208–210] and increase the weight of weaned piglets [19], as well as regulate piglet immune status by increasing the content of n-3 PUFAs in immune cells and lowering the synthesis of proinflammatory eicosanoids [211]. Additionally, n-3 functional fatty acids can improve glucose absorption in the piglet jejunum and maintain intestinal stability [212]. Supplementing short- and medium-chain functional fatty acids in the diet of sows during the lactation period also has positive effects. As reported, adding butyric acid during the lactation period can enhance the acetylation of H3K27 in piglet skeletal muscle and increase the expression of PPARγ, thereby affecting lipid metabolism in piglet skeletal muscle and improving piglet growth [213]. During the lactation period, adding medium-chain functional fatty acids like lauric acids has a substantial antibacterial effect and boosts immunity in sows and piglets by preventing harmful bacterial growth [214]. According to a comprehensive comparative study, adding butyric acid to the diet during the lactation period had a stronger influence on intestinal health than adding medium-chain fatty acids or n-3 functional fatty acids, with a greater decline in piglet preweaning mortality [176]. The addition of medium-chain fatty acids to the diet, on the other hand, decreased the duration between weaning and estrus more significantly than butyric acid or n-3 functional fatty acids. N-3 functional fatty acids added to diet increase the fat and protein contents in colostrum to the greatest extent. The above studies demonstrate that there are differences in the way different types of functional fatty acids work during the lactation period. There is a lack of more research clarifying the use of various types of functional fatty acids in sows during the lactation period, but systematic and reasonable fatty acid nutrition is highly necessary for sows during the lactation period.

### Role of functional fatty acids in dairy cows during the lactation period

Unlike sows, cows in the lactation period are tasked with milk production in enormous quantities to meet human demand for dairy products while meeting the nutritional needs of 1 or 2 calves, and the major goal of cow production is to produce high-yield and high-quality milk for human. Therefore, high-yielding cows have greater energy requirements beyond their ability to absorb energy from diet [215]. Moreover, corn silage-based diets have replaced pasture feeding in modern cows, and the predominant source of fatty acids has transitioned from ALA to LA [216]. As a result of reduced pasture feeding, the amount of conjugated LA produced by rumen biohydrogenation has also decreased. Adding fatty acids to diet can boost energy density without increasing rumen acid production [217]. The 2 primary products to minimize the effect of fat on rumen fermentation are extruded saturated free fatty acids and calcium salts of unsaturated fatty acids. However, the calcium salts of unsaturated fatty acids are not fully protected in the rumen, and calcium ion decomposition facilitates the biohydrogenation of unsaturated fatty acids in the rumen [218]. The focus of research has been on improving the physicochemical properties of fatty acid supplements to meet the fatty acid requirements of cows, and it is now widely accepted that saturated fatty acid intake should be reduced and polyunsaturated functional fatty acid intake should be increased, especially the intake of n-3 functional fatty acids [190], which is important for the health of humans who rely on dairy products. Due to the fact that in contrast to primitive societies, the modern human diet is rich in saturated fatty acids and n-6 PUFAs but deficient in n-3 PUFAs, and n-3 PUFA deficiency is linked to the development of coronary heart disease and other noninfectious disorders [219, 220]. Compared with those from placental transfer, fatty acids from colostrum or milk, on the other hand, have a stronger influence on calves’ development and health [221, 222]. Furthermore, in a recent study, n-3 functional fatty acids in colostrum have been found to have favorable benefits on calf inflammatory responses [223]. In both cows and calves, adding ALA, either alone or in combination with LA, to diet can boost both average daily gains and feed efficiency [221, 224].

## Summary

In conclusion, livestock can obtain functional fatty acids through feed and endogenous synthesis, these fatty acids play important roles throughout the reproductive cycle of livestock. The role of functional fatty acids in livestock reproduction is affected by the carbon chain length and degree of unsaturation of the fatty acids. Through continuous improvement of experimental techniques and analytical methods, some key mechanisms of functional fatty acids have been elucidated. Fatty acids can provide energy for oocyte and embryo development through β-oxidation, and they also play a role in the synthesis of lysophosphatidic acid, PG and steroid hormones, thereby influencing early embryo development and embryo implantation. Fatty acids are also closely associated with placental vascular development, lactation performance, milk quality and young livestock development. However, the requirements of livestock for fatty acid nutrition during the reproductive process are dynamic and diverse, but these research results are based on single fatty acids; the ideal fatty acid requirements for each stage of reproduction are unknown.

Obviously, enhancing livestock reproductive performance is a challenge involving the entire reproductive cycle, with close and intricate links between stages, and negative effects at any stage will have a long-term impact on the entire reproductive process. Therefore, it is important to reveal the regulation function of fatty acids and their metabolites in each key stage of the reproductive cycle. Currently, the researches on ideal fatty acid mode are blank. It has great significance to establish an ideal fatty acid pattern for specific stages to improve reproductive potential in livestock. The ideal fatty acid model requires systemic researches to elucidate the order of importance and the ratio of different fatty acids or the relevant derivatives, as well as the related influences on the performance and physiological function in livestock.

## Data Availability

The datasets used and/or analyzed during the current study are available from the corresponding author on reasonable request.

## Abbreviations

ACP: Acyl carrier protein
ALA: α-linolenic acid
ARA: Arachidonic acid
CoA: Coenzyme A
COCs: Cumulus-oocyte complexes
DHA: Docosahexaenoic acid
EPA: Eicosadienoic acid
ER: Estrogen receptor
FF: Follicular fluid
LA: Linoleic acid
LCPUFAs: Long-chain polyunsaturated fatty acids
MAPK: Mitogen-activated protein kinase
NEFAs: Nonesterified fatty acids
OA: Oleic acid
PGs: Prostaglandins
PPARγ: Peroxisome proliferator-activated receptor gamma
PR: Progesterone receptor
PTGS: Prostaglandin-endoperoxide synthase
PUFAs: Polyunsaturated fatty acids
